# Caregiver Stress and Empathy: Neural and Hormonal Correlates

**DOI:** 10.1093/geroni/igab046.677

**Published:** 2021-12-17

**Authors:** Shalmali Mirajkar, David Warren, Janelle Beadle

**Affiliations:** 1 Saint Louis University, St. Louis, Missouri, United States; 2 Department of Neurological Sciences, University of Nebraska Medical Center, Omaha, Nebraska, United States; 3 University of Nebraska at Omaha, Omaha, Nebraska, United States

## Abstract

Providing care to older adults with chronic conditions can be emotionally meaningful and stressful. The tend-and-befriend theory highlights the role of affiliation/empathy in stress reduction, but it has not been established whether this theory extends to caregivers for older adults. Addressing this gap, we assessed caregiver empathy and stress through behavioral, hormone, and neuroimaging measures. In Experiment 1, we compared 19 caregivers (Mage=67.1) to 24 non-caregivers (Mage=72.6), and found that caregivers with a greater reduction in cortisol to an empathic context showed greater prosocial behavior (r2=0.3). In experiment 2 (N=32), we examined differences between caregivers and non-caregivers in whole brain resting-state functional connectivity (RSFC) with seed regions of interest (posterior cingulate cortex (PCC); amygdala), and covariation of RSFC with empathy (α=0.05). For emotional empathy, caregivers had stronger connectivity between the PCC seed, medial prefrontal cortex, and right supramarginal gyrus, and between the amygdala seed and the right middle frontal gyrus.

